# A Directed Acyclic Graph-Large Margin Distribution Machine Model for Music Symbol Classification

**DOI:** 10.1371/journal.pone.0149688

**Published:** 2016-03-17

**Authors:** Cuihong Wen, Jing Zhang, Ana Rebelo, Fanyong Cheng

**Affiliations:** 1 College of electrical and information engineering, Hunan University, Changsha, China; 2 INESC Porto, Universidade do Porto, Porto, Portugal; Qom University, ISLAMIC REPUBLIC OF IRAN

## Abstract

Optical Music Recognition (OMR) has received increasing attention in recent years. In this paper, we propose a classifier based on a new method named Directed Acyclic Graph-Large margin Distribution Machine (DAG-LDM). The DAG-LDM is an improvement of the Large margin Distribution Machine (LDM), which is a binary classifier that optimizes the margin distribution by maximizing the margin mean and minimizing the margin variance simultaneously. We modify the LDM to the DAG-LDM to solve the multi-class music symbol classification problem. Tests are conducted on more than 10000 music symbol images, obtained from handwritten and printed images of music scores. The proposed method provides superior classification capability and achieves much higher classification accuracy than the state-of-the-art algorithms such as Support Vector Machines (SVMs) and Neural Networks (NNs).

## Introduction

For centuries, musical scores have been preserved in libraries and museums and made available as original manuscripts or scanned copies. The propagation and availability of such musical sources are limited by the storage methods. Fortunately, with the development of scanning and pattern recognition technology, digital libraries have become increasingly popular. Over the last few years, a growing amount of information has been obtained from digital libraries or the internet. Therefore, the transformation from traditional music sheets to a machine readable format is essential. For decades, many efforts have been devoted to the development of OMR systems. Unfortunately, the currently available music recognition methods are far from satisfactory.

**Fig 1:**
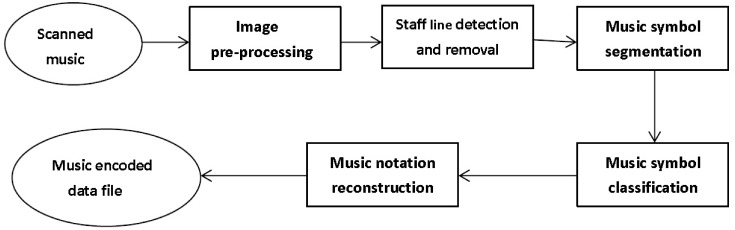
A general architecture for OMR.

Technically, the OMR could be considered an extension of the Optical Character Recognition (OCR). An OMR system typically encompasses five main steps: image preprocessing, staff line detection and removal, music symbol segmentation, music symbol classification, and music notation reconstruction, which can be seen in Fig 1.

The remainder of this paper is structured as follows. In Section 2, the related works on this area are reviewed. In Section 3, the proposed algorithm for DAG-LDM is presented. In Section 4, the experiment settings are described. Section 5 discusses the performance obtained using the proposed method and compares it with that of other state-of -the-art approaches. We summarize our conclusions in the last section.

## Related works

Recently, people have been paying more and more attention to the music symbol classification step. [1, 2]performed particularly important early work of this kind using Neural Networks (NNs). But they focused on only a few classes of the music symbols. [3] developed an algorithm to learn a Mahalanobis distance for the k-NNs and extended it to SVMs. However the classification accuracy was below 80% In some instances, the operation of symbol classification was linked to the segmentation of objects from the music symbols. For example, segmentation and classification were performed simultaneously using HMM without removing the staff lines in [4]. The algorithm was valuable for the global feature extraction and capable of distinguishing symbols or not symbols. But the symbols from different classes were not classified successfully. In [5], they compared the results of different algorithms such as Support Vector Machines(SVMs), Neural Networks (NNs), k-Nearest Neighbors(k-NNs) and Hidden Markov Models(HMMs). The performances of these methods were compared using both real and synthetic scores. A more recent study for classification was based on Combined Neural Network [6], which had a higher accuracy than NNs. But both of the works suffer from unsatisfactory recognition rates, especially for handwritten music symbols.

Although all the above mentioned approaches have been demonstrated to be effective in specific environments, the classification accuracy still requires improvement. Zhang etc.[7, 8] proposed the Large margin Distribution Machine(LDM) in 2014. They proved that the binary classification capability of the LDM is better than the SVM using big datasets. LDM optimizes the margin distribution by maximizing the margin mean and minimizing the margin variance simultaneously. The distribution of the music symbol samples and the margin distribution are crucial during the classification process. Therefore, we consider using the LDM to classify the multi-class music symbols. In this paper, we extend the LDM to DAG-LDM for the music symbol classification. The performance is improved using the proposed algorithm

### Proposed multi-class LDM named DAG-LDM

**Fig 2:**
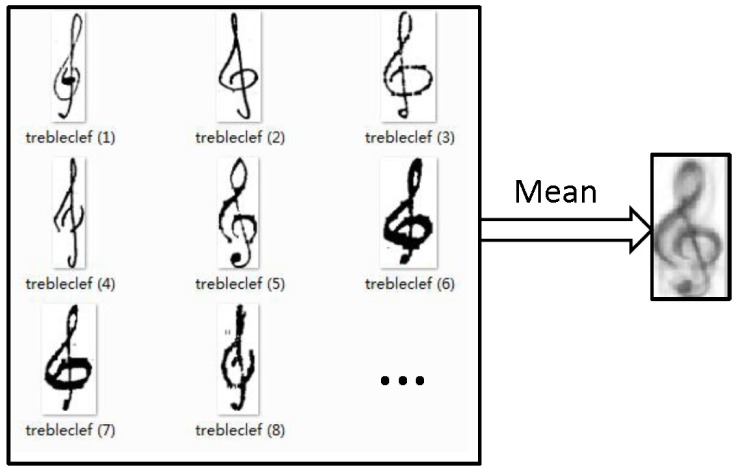
The contour mean image.

Before presenting the theory of the Large Margin Distribution Machine (LDM), it is instructive to motivate the concept applied to music symbol classification by analyzing the features of music symbols. We consider a database of 295 treble clef samples and the contour mean image—see Fig 2. The distribution of the samples and the margin distribution are crucial during the classification process, especially when dealing with handwritten symbols. Because the LDM optimizes the margin mean and the margin variance, it is a good choice in this context.

We denote the data set space by **x** *ϵR*^*d*^ and the label set by *y* = {+1,−1}. D is an unknown distribution over*x*×*y*. A training set of size m can be expressed as *S* = {(**x**_**1**_, *y*_1_), (**x**_**2**_, *y*_2_), …(**x**_**m**_, *y*_*m*_)}, and it is drawn identically and independently(i.i.d.) according to the distribution D. The SVMs and LDM learn a function to predict the labels of the test samples.

In the SVM, a linear model is designed as

$$\begin{array}{c} f\left(x\right)=\omega^{T}\phi \left(x\right) \end{array}$$ (1)

where ***ω*** is a linear predictor, *ϕ*(**x**) is a feature mapping of induced **x** by a kernel *k*, i.e., *k*(**x**_*i*_,**x**_*j*_) = *ϕ*(**x**_*i*_)^*T*^ *ϕ*(**x**_*j*_). According to [9, 10], the margin of instance(**x**_*i*_, *y*_*i*_) is formulated as,

$$\begin{array}{c} \gamma_{i}=y_{i}\omega^{T}\phi \left(x_{i}\right),\forall i=1,...,m. \end{array}$$ (2)

As shown in [11], the hard-margin SVM can be formulated as

$$\begin{array}{ccc} & & \min_{w}\frac{1}{2}\omega^{T}\omega \\ & & s.t.y_{i}\omega^{T}\phi \left(x_{i}\right)\geq 1,i=1,...,m. \end{array}$$ (3)

Regarding the non-separable cases, where the training examples cannot be separated with a zero error, a soft-margin SVM was discussed in [12].

$$\begin{array}{ccc} & & \min_{w,\xi }\frac{1}{2}\omega^{T}\omega +C\sum_{i=1}^{n}\xi_{i}\\ & & s.t.y_{i}\omega^{T}\phi \left(x_{i}\right)\geq 1-\xi_{i}\\ & & \xi_{i}\geq 0,i=1,...,m. \end{array}$$ (4)

where ***ξ*** = [*ξ*_1_, …, *ξ*_*m*_] is a slack variable, and C is a constant that penalizes the training errors. Therefore, the SVM only considered the single-point minimum margin and did not exploit the whole margin distribution. As we know, the mean and the variance of the margin are the two most essential statistics for characterizing the margin distribution. According to the definition in Eq (2), the mean margin is

$$\begin{array}{c} \gamma \bar{}=\sum_{i=1}^{n}y_{i}\omega^{T}\phi \left(x_{i}\right)=\frac{1}{m}{\left(\text{Xy}\right)}^{T}\omega , \end{array}$$ (5)

and the margin variance can be expressed as

γ^=1m2∑i=1m∑j=1m(yiωTϕ(xi)-yjωTϕ(xj))2=2m2(mωTXXTω-ωTXyyTXTω). (6)

The LDM adopts the maximal margin mean and minimum margin variance simultaneously. Thus, the soft-margin LDM can be formulated as

minw,ξ12ωTω+λ1γ^-λ2γ¯+C∑i=1nξis.t.yiωTϕ(xi)≥1-ξiξi≥0,i=1,...,m. (7)

The optimal object function is expressed using the following quadratic programming problem

$$\begin{array}{ccc} & & \min_{w,\xi }\frac{1}{2}\omega^{T}\omega +\frac{2\lambda_{1}}{m^{2}}\left(m\omega^{T}{\text{XX}}^{T}\omega -\omega^{T}{\text{Xyy}}^{T}X^{T}\omega \right)\\ & & -\lambda_{2}\frac{1}{m}{\left(\text{Xy}\right)}^{T}\omega +C\sum_{i=1}^{n}\xi_{i}\\ & & s.t.y_{i}\omega^{T}\phi \left(x_{i}\right)\geq 1-\xi_{i}\\ & & \xi_{i}\geq 0,i=1,...,m \end{array}$$ (8)

**Fig 3:**
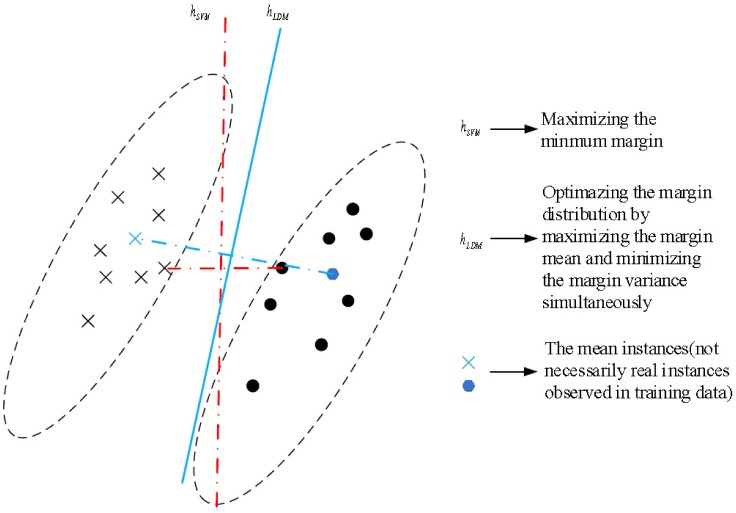
A simple illustration of SVM and LDM.

From Eq (8) we can intuitively find that LDM is superior to SVM because SVM is a special example of LDM when *λ*_1_ = *λ*_2_ = 0. The values of *λ*_1_, *λ*_2_ are derived from cross-validation. A simple illustration for SVM and LDM is shown in Fig 3.

Like the SVM, there are three common types of inner-product kernels for LDM: linear kernel, polynomial kernel, and radial-basis function. Here we adopt a radial-basis function for the model

$$\begin{array}{c} k\left(x,x_{i}\right)=exp(-g∥x-x_{i}{∥}^{2}),g>0. \end{array}$$ (9)

The LDM was initially proposed for the binary classification problem and cannot be used for the multi-class problems directly. Hence, we generalize the LDM to the DAG-LDM.

Usually, the multi-class classification problem is solved using a decomposition to several binary problems for which the standard LDM can be used. For example, in the SVMs, one-against-rest (1-v-r) and one-against-one(1-v-1) are often applied. For the 1-v-r method, the classification problem to k classes is decomposed to k binary decisions. The ith SVM will be trained with all of the samples belonging to the i-th class with positive labels, and all other samples with negative labels. The result for a test sample is the class that corresponds to the SVM with the highest output value. A significant disadvantage of the 1-v-r approach is that the training is time consuming. The 1-v-1 method, constructs all of the possible two-class classifiers from a training set of N classes, each classifier being trained on only two out of N classes. The final decision is chosen by majority vote. Unfortunately, there is no bound on the generalization error for both methods. To avoid these disadvantages, we adopt a learning architecture named DAG [13] to combine many 1-v-1 LDMs into a multi-class LDM.

**Fig 4:**
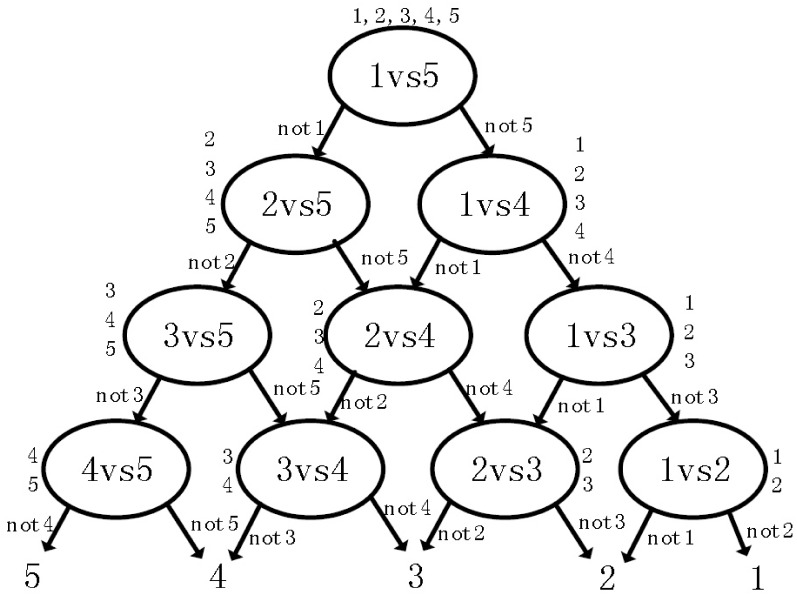
A simple illustration of DAG for a 5-class problem.

Here, we introduce a novel algorithm for multi-class classification, which places the 1-v-1 LDM into the nodes of a DAG. This algorithm, named DAG-LDM, is efficient for training and evaluation. The edges of a DAG have an orientation and no cycles. For a N-class problem, there are N(N-1)/2 nodes and N leaves. Each node stands for a classifier and has two arcs leaving it. The nodes are arranged in a triangle with the single root node at the top, two nodes in the second layer and so on until the final layer of N leaves. A class list is initialized with a list of all classes for the top node. The top classifier is trained using the examples from the first and last classes of the list. When given a test sample, if the top node prefers one of the two classes, the other class is eliminated from the list. Then, the DAG-LDM proceeds to test the first and last elements of the new list. In order to achieve a decision for a test sample, N-1 decision nodes will be evaluated. Fig 4 is a simple illustration of a DAG for a 5-class problem.

In terms of the choice of the class order in the DAG list, we arrange the order manually according to the difference among the classes. Specifically, we place the classes that are easier distinguished in the upper layers. This reduces the possibility of misclassification in the upper layers.

## Experiments

We applied the proposed DAG-LDM to both handwritten and printed music symbol data sets, respectively. In Section 4.1, we provide a description of the data sets, while in Section 4.2, we briefly describe the details of the training. In Section 4.3, the parameter searches are studied.

### Database

A data set of both handwritten scores and printed scores is used to perform the DAG-LDM. The real scores consist of 6 handwritten scores from 6 different composers, with the ground truth obtained manually. In the scanned data set, there are 9 scores available from the data set of [14], written in the standard notation. A number of distortions are applied to the scanned scores. The deformations applied to these scores are curvature, rotation, Kanungo and white speckles, −see [14] for more details. After the deformations, we have a total of 45 scanned images. Finally, more than 10000 music symbols are generated from 51 music scores.

**Fig 5:**
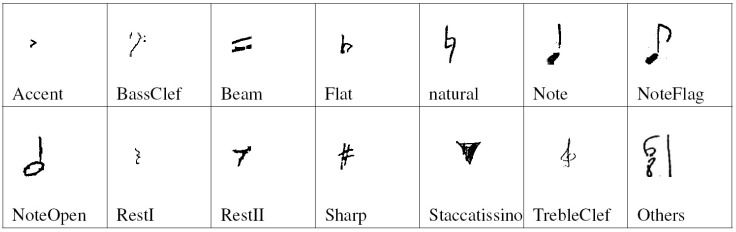
Full set of the handwritten music symbols considered.

**Fig 6:**
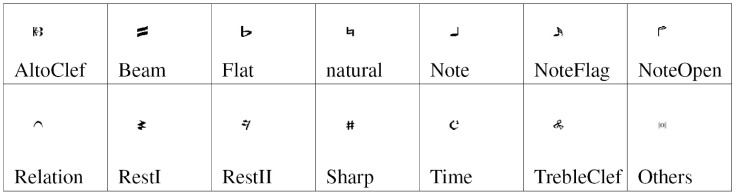
Full set of the printed music symbols considered.

Each image of a music symbol was previously binarized with the Otsu threshold algorithm [15]. Then, the images are resized to 60*20 pixels and converted to a vector of 1200 binary values. We choose this size according to the shapes of the symbols: the most common has the height greater than the width, such as notes, and notes flags. Please see Figs 5 and 6.

### Experimental design

The training of the networks is performed in MATLAB 2014a. The data sets are divided into 14 classes according to their shape as shown in Fig 5 for handwritten symbols and Fig 6 for printed symbols. For each class, half of the examples are randomly selected as the training data, and the remaining examples are used as the testing data.

The classifiers of the DAG are obtained by training each ij-node only on the subset of training points labeled as i or j. As both the handwritten and printed music symbols are classified into 14 classes, we should train 14*13/2 = 91 classifiers.

The best parameters for each model are found based on a ten-fold cross-validation scheme that is conducted on the training set. This procedure randomly divides a data set into ten disjoint folds of approximately equal size, and each fold is in turn used to test the model induced from the other nine folds by a classification algorithm [16]. The performance of the classification algorithm is evaluated by the average of the k accuracies resulting from ten-fold cross validation, and hence the level of averaging is assumed at fold. Finally, the error of the model is estimated on the test set. In our experiment, we search for the best parameters of *λ*_1_, *λ*_2_, *C* in Eq (8) and *g* in Eq (9) using cross-validation.

## Results and Discussion

The handwritten and printed music symbols are randomly split into training and test sets. We repeat this procedure 10 times to obtain more stable results for the average accuracy. A confidence interval is computed for the mean average accuracies of ten tests as

$$\begin{array}{c} \bar{X}-t^{*}\frac{S}{\sqrt{N}}\leq \mu \leq \bar{X}+t^{*}\frac{S}{\sqrt{N}} \end{array}$$ (10)

where t* is the upper (1-C)/2 critical value for the t distribution with N-1 degrees of freedom, and $\bar{X}$ is the mean of the ten average accuracies, S is the standard deviation and N = 10.

Neural network(NN) is another powerful classification model. The inspiration for the neural network came from the examination of animals’ central nervous systems. In this work, a specific architecture of neural networks was exclusively used, namely the multi-layer perceptron (MLP), one type of a feed-forward network [17]. A MLP is a layered structure consisting of nodes or units (called neurons) and one-way connections or links between the nodes of successive layers. The training of the networks was carried out under Matlab 2014a and was done using back-propagation together with the Levenberg-Marquardt algorithm. We use a network with K outputs, one corresponding to each class, and target values of 1 for the correct class and 0 otherwise.

The theory of SVM has been described above. In this work, a radial-basis function network was used. The binary classifier can be extended to multi-class scenarios. Of the multiple extensions available in the literature, we used the one against one methodology.

Table 1 presents the results obtained applying the proposed DAG-LDM, the SVM [5] and the NN [5] classifiers to the handwritten database.

**Table 1:** The results of DAG-LDM and SVMs and NNs for the handwritten music symbols.

Classes	Accuracy NNs(%)[5]	Accuracy of SVMs (%)[5]	Accuracy of DAG-LDM (%)
Accent	85	99	100
BassClef	13	77	96
Beam	85	95	93
Flat	84	98	100
Natural	98	98	100
Note	82	96	95
NoteFlag	51	89	94
NoteOpen	3	40	96
RestI	78	97	100
RestII	96	100	100
Sharp	85	98	98
Staccatissino	58	100	94
TrebleClef	40	90	98
Others	52	89	94
99%CI for the expected performance in percentage:Average	[81;84]	[95;96]	[96.1;97.3]

The first assessment is that within the DAG-LDM methodology, an overall improvement was observed, with a 99% confidence interval for the expected performance [96.1%;97.3%]. Interestingly, 100 percent was achieved for 5 classes, which is a superior result compared to those for the other methods. Moreover, although the SVMs performed well in terms of average accuracy, they exhibited strong difficulties with some classes, presenting very low accuracy values. The lowest accuracy using DAG-LDM is the one for Beam(93%), which is much higher than 40% for NoteOpen using SVMs. Our approach of DAG-LDM seems to be a promising methodology for the purpose of music symbol classification.

The results obtained for the printed music symbols are shown in Table 2. They further support the superiority of the DAG-LDM model, with a 99% confidence interval for the expected performance [98.1%;99.6%]. As expected, all the three models perform better when processing printed symbols.

**Table 2:** The results of DAG-LDM and SVMs and NNs for the printed music symbols.

Classes	Accuracy of NNs(%)[5]	Accuracy of SVMs (%)[5]	Accuracy of DAG-LDM (%)
AltoClef	94	98	99.5
Beam	92	100	100
Flat	97	99	100
Natural	94	100	100
Note	90	99	98.6
NoteFlag	70	96	97.4
NoteOpen	88	97	98.8
Relation	55	87	100
RestI	85	100	100
RestII	75	100	99.7
Sharp	97	100	100
Time	40	100	99.2
TrebleClef	93	100	100
Others	65	93	96.5
99%CI for the expected performance in percentage:Average	[88;89]	[97;99]	[98.1;99.6]

## Conclusions

In this paper, a DAG-LDM classifier is applied towards the recognition of music symbols. Considering that the margin distribution is to a certain extent accordant with the mean instances, we adopt the margin distribution rather than a single minimum margin in our model. Each classifier inside the DAG-LDM maximizes the margin mean and minimizes the margin variance simultaneously. As has been shown in the test, significant classification improvements are obtained, especially when processing handwritten symbols. The proposed DAG-LDM approach is more efficient and less time consuming than the 1vr and 1v1 methods. Furthermore, the proposed algorithm has a broad range of applications in solving other multi-class problems. One possible weakness is that when the shapes of the samples are diversiform, such as Beams, NoteFlags, the classification performance is not so satisfied. Future investigations could include applying other classification models to music symbol recognition, e.g., Deep Learning method [18]. It is also worthwhile to study the proposed LDM with other efficient multi-class algorithms for SVMs, e.g., the algorithm based on the SVM-BTA [19].

## Supporting Information

S1 File The dataset of the music sheets. The real scores consist of 6 handwritten scores from 6 different composers. In the scanned data set, there are 9 scores available from the data set of [14], written in the standard notation.
(ZIP)
