# Supplementary figures and images for: A Directed Acyclic Graph-Large Margin Distribution Machine Model for Music Symbol Classification
Source: PLoS One. 2016 Mar 17;11(3):e0149688. doi: 10.1371/journal.pone.0149688 (PMC4795649; doi:10.1371/journal.pone.0149688)

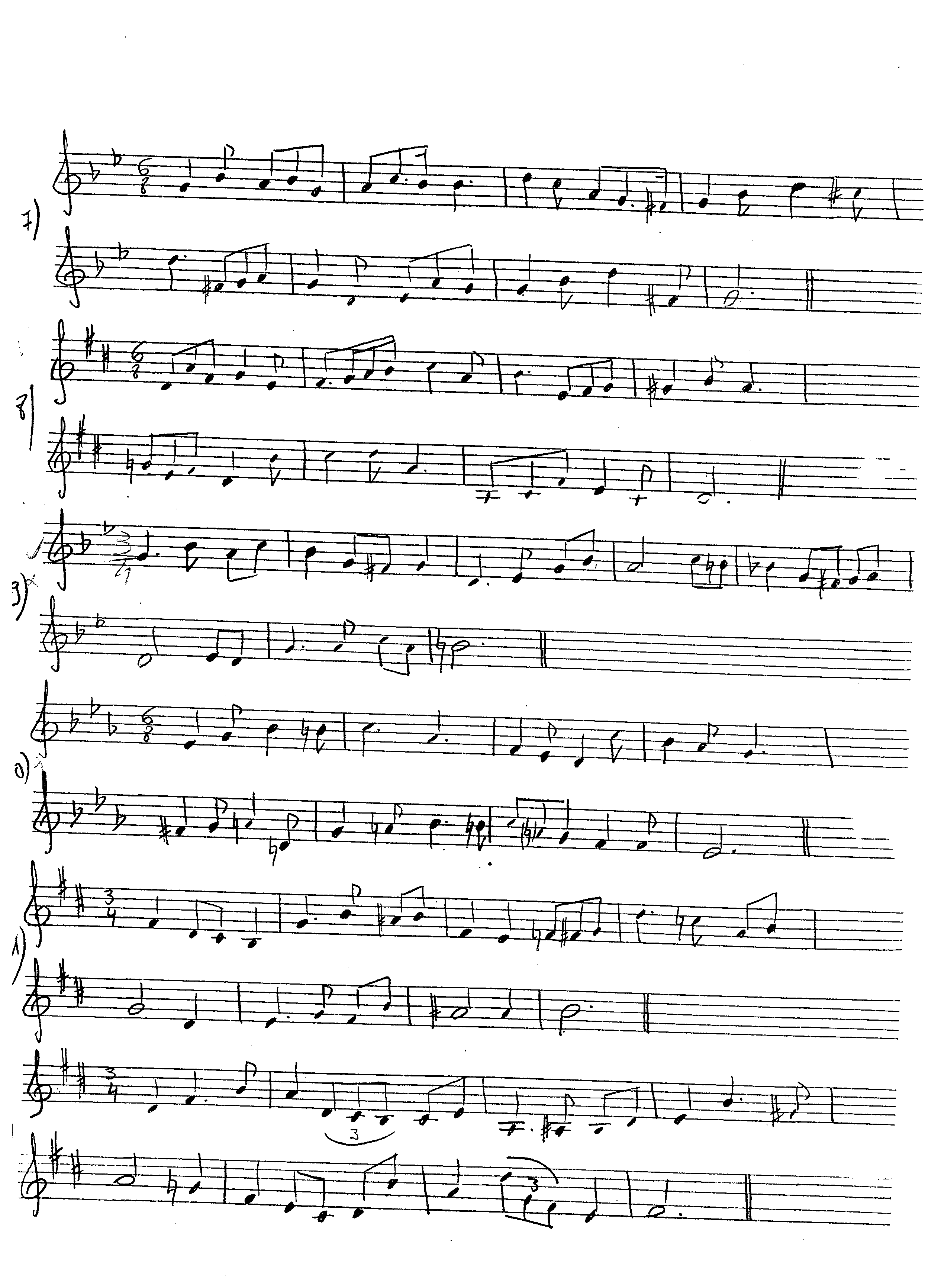

Supplement: S1 File — The real scores consist of 6 handwritten scores from 6 different composers. In the scanned data set, there are 9 scores available from the data set of [14], written in the standard notation. (ZIP) [file pone.0149688.s001.zip › DATABASE/IMAGES_REAL/img009.png]

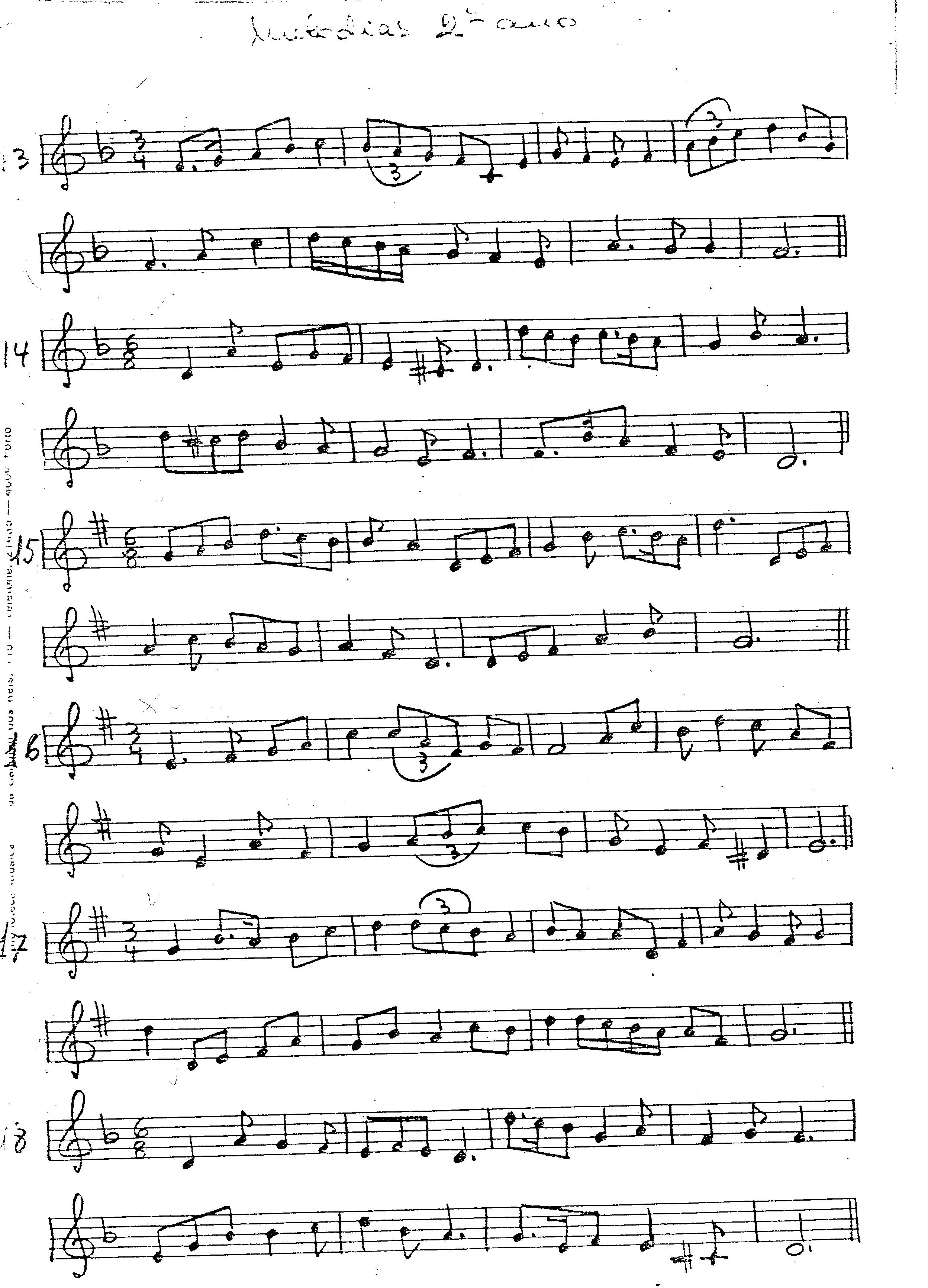

Supplement: S1 File — The real scores consist of 6 handwritten scores from 6 different composers. In the scanned data set, there are 9 scores available from the data set of [14], written in the standard notation. (ZIP) [file pone.0149688.s001.zip › DATABASE/IMAGES_REAL/img010.png]

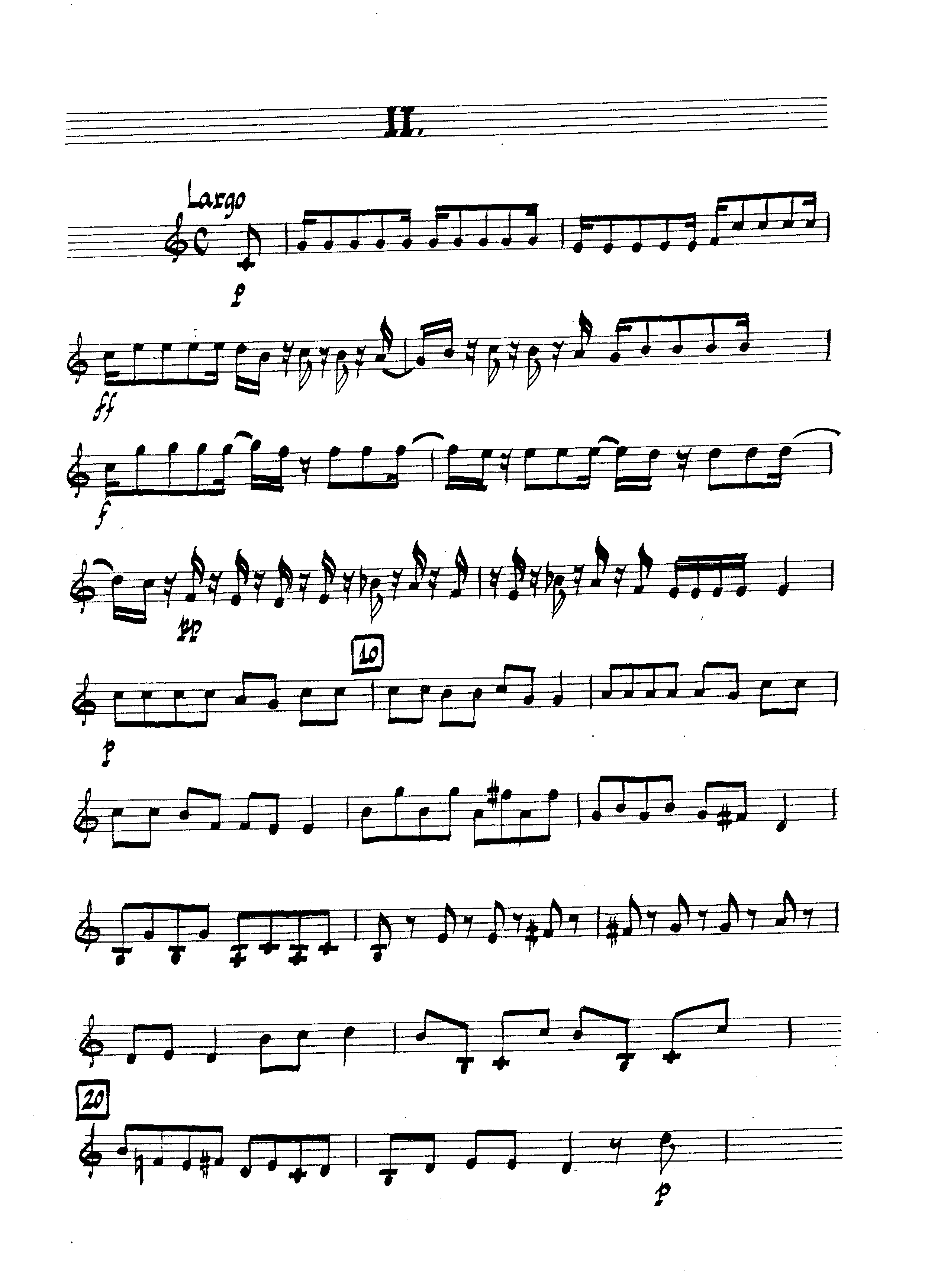

Supplement: S1 File — The real scores consist of 6 handwritten scores from 6 different composers. In the scanned data set, there are 9 scores available from the data set of [14], written in the standard notation. (ZIP) [file pone.0149688.s001.zip › DATABASE/IMAGES_REAL/img014.png]

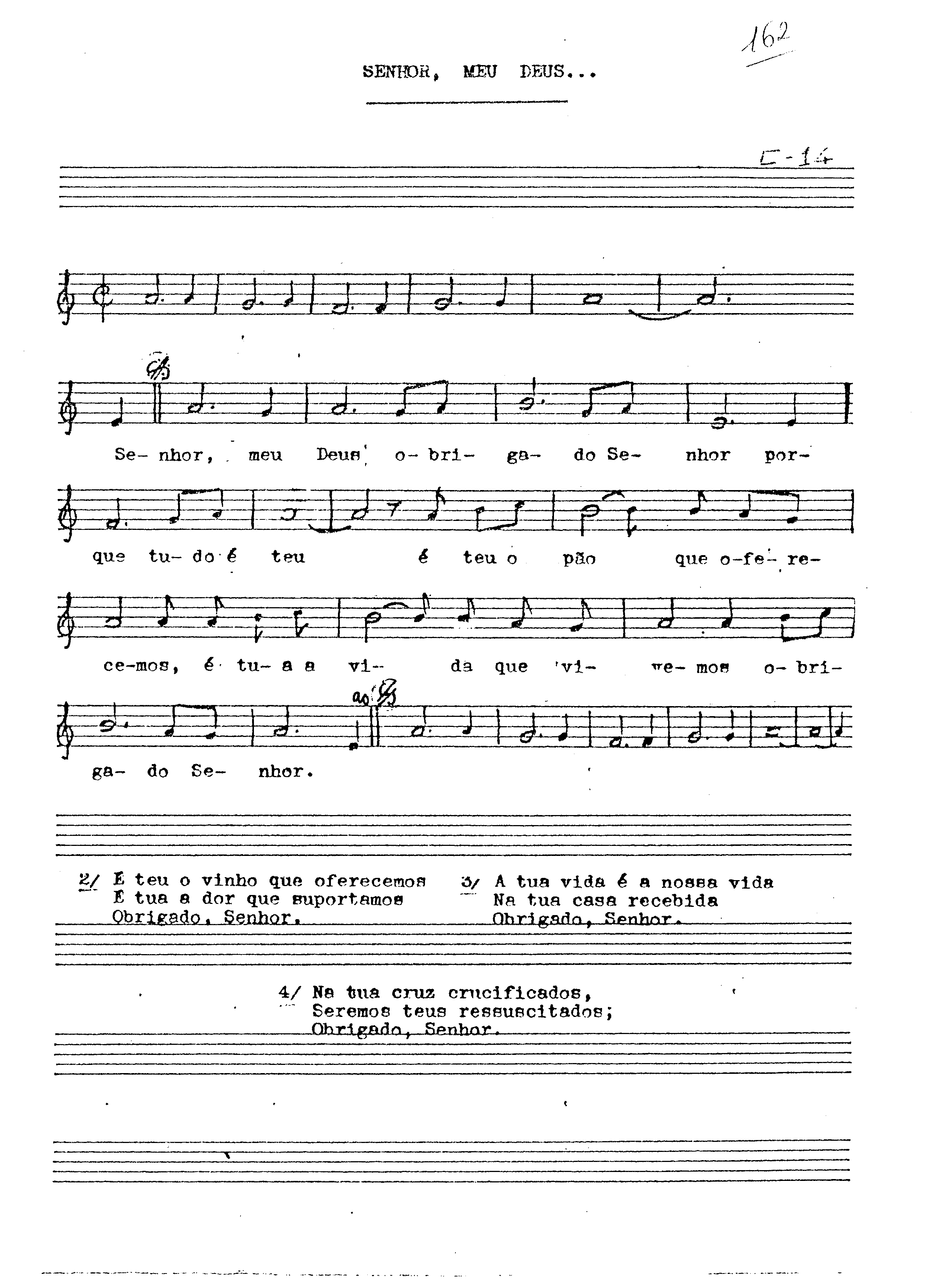

Supplement: S1 File — The real scores consist of 6 handwritten scores from 6 different composers. In the scanned data set, there are 9 scores available from the data set of [14], written in the standard notation. (ZIP) [file pone.0149688.s001.zip › DATABASE/IMAGES_REAL/img033.png]

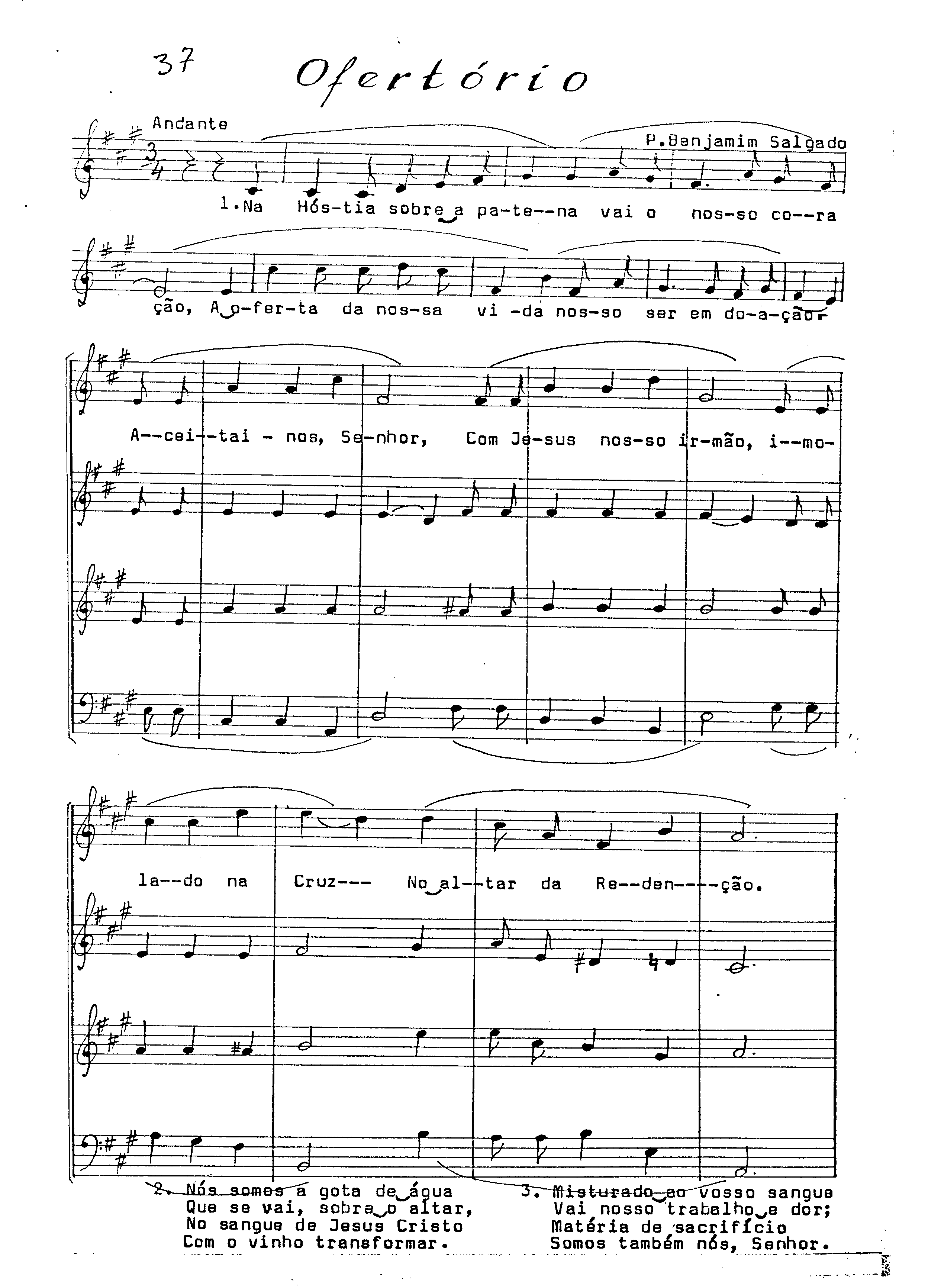

Supplement: S1 File — The real scores consist of 6 handwritten scores from 6 different composers. In the scanned data set, there are 9 scores available from the data set of [14], written in the standard notation. (ZIP) [file pone.0149688.s001.zip › DATABASE/IMAGES_REAL/img036.png]

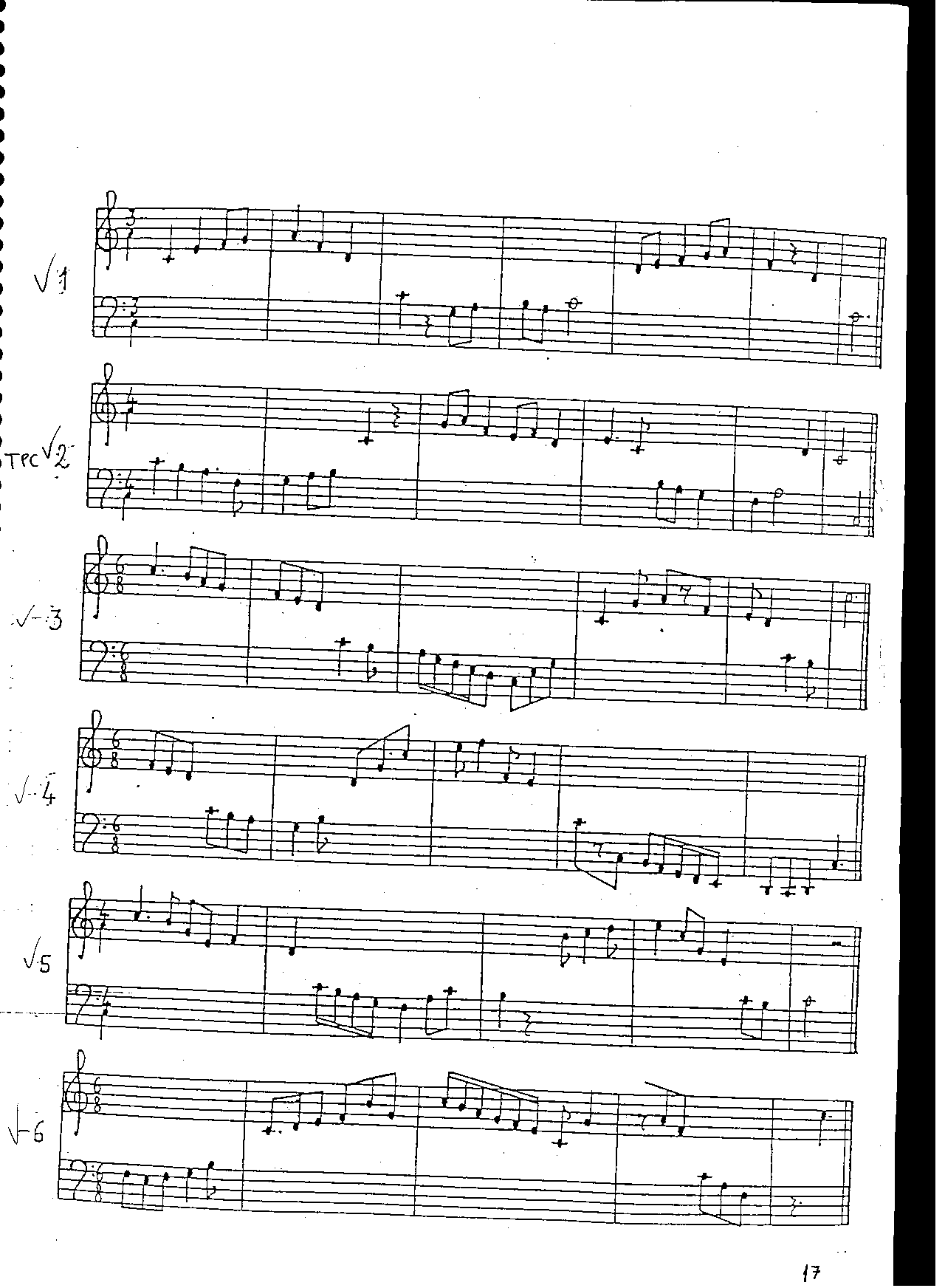

Supplement: S1 File — The real scores consist of 6 handwritten scores from 6 different composers. In the scanned data set, there are 9 scores available from the data set of [14], written in the standard notation. (ZIP) [file pone.0149688.s001.zip › DATABASE/IMAGES_REAL/img46.png]

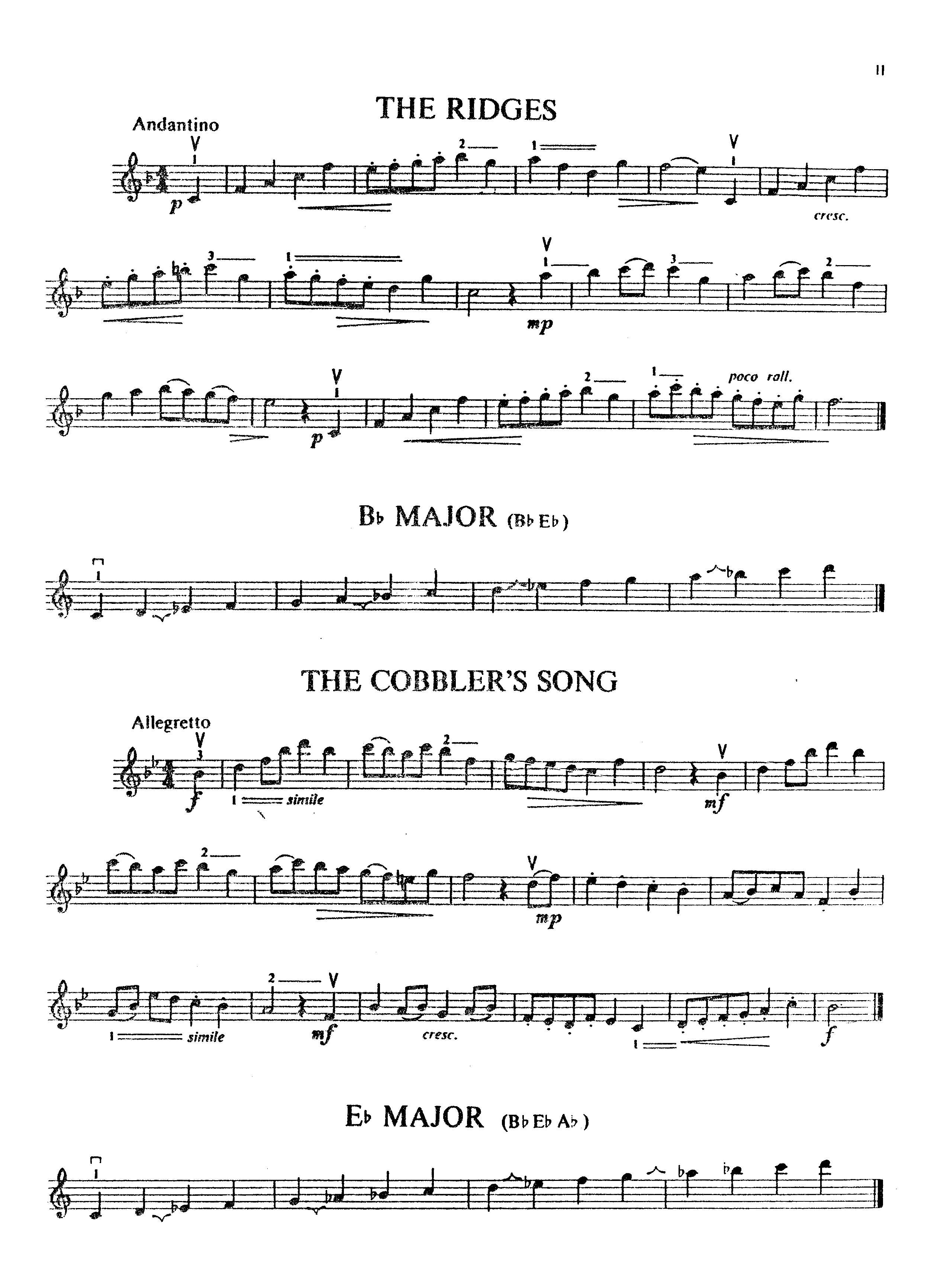

Supplement: S1 File — The real scores consist of 6 handwritten scores from 6 different composers. In the scanned data set, there are 9 scores available from the data set of [14], written in the standard notation. (ZIP) [file pone.0149688.s001.zip › DATABASE/imgs_SCANED/img004.png]

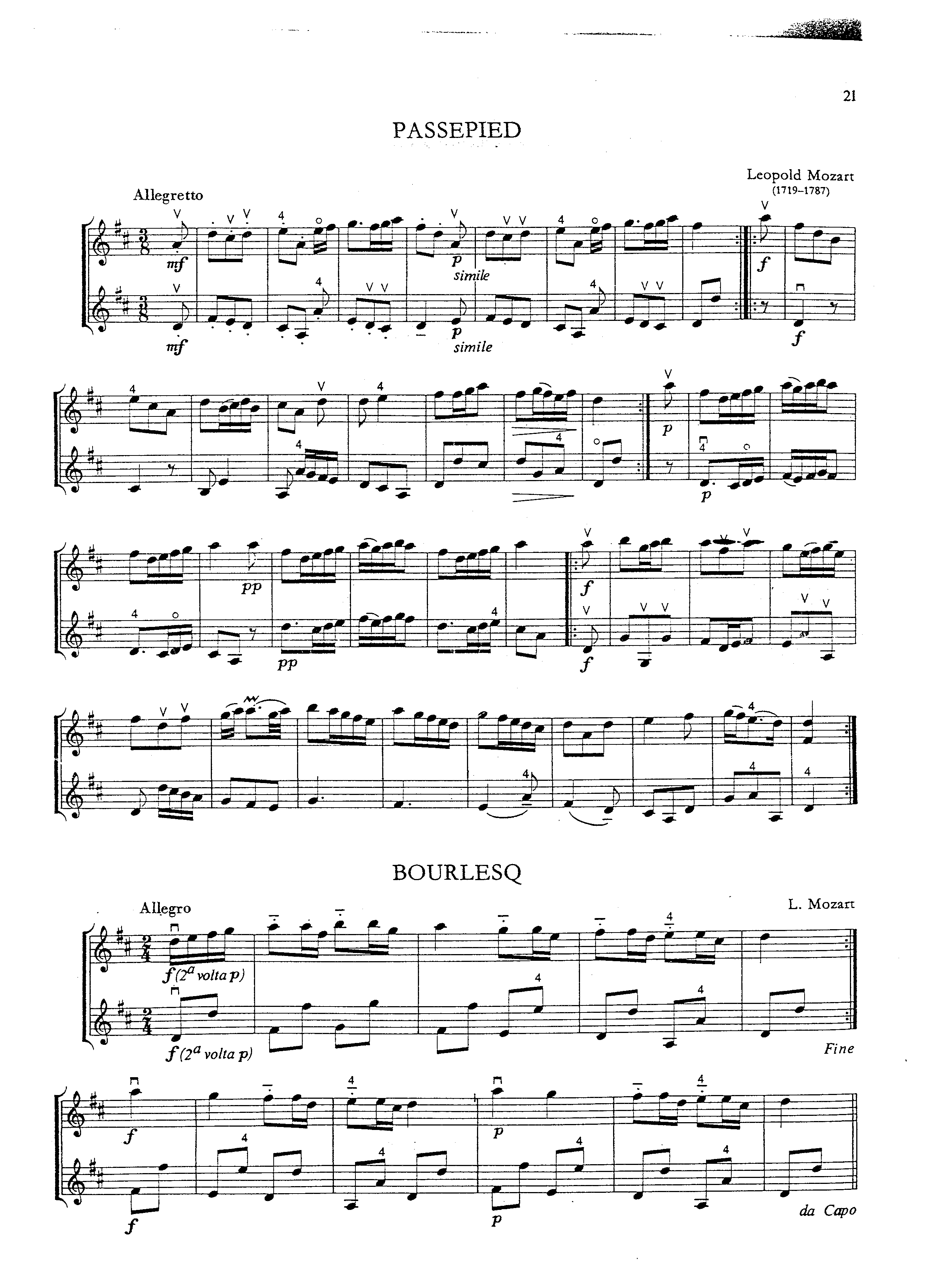

Supplement: S1 File — The real scores consist of 6 handwritten scores from 6 different composers. In the scanned data set, there are 9 scores available from the data set of [14], written in the standard notation. (ZIP) [file pone.0149688.s001.zip › DATABASE/imgs_SCANED/img006.png]

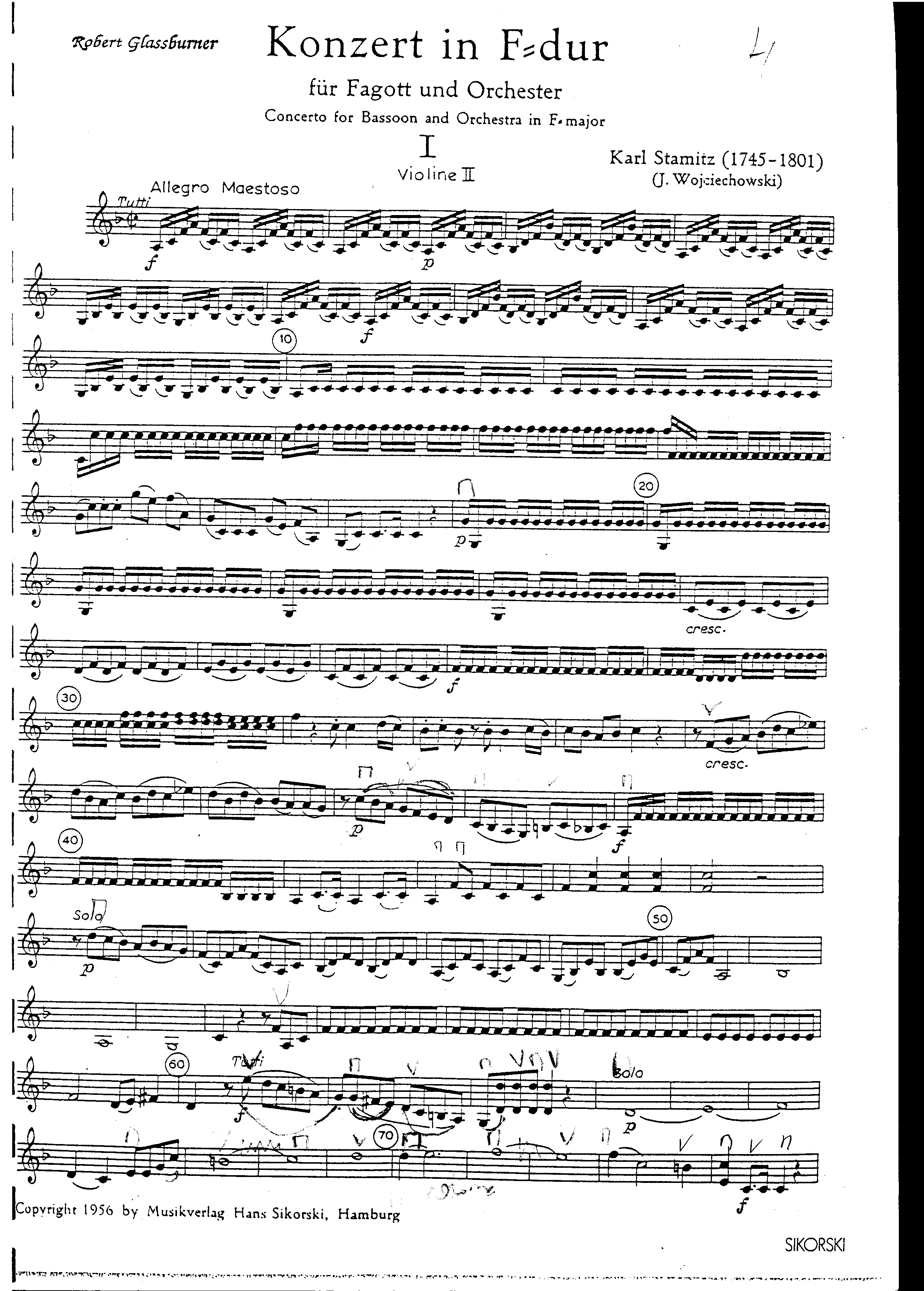

Supplement: S1 File — The real scores consist of 6 handwritten scores from 6 different composers. In the scanned data set, there are 9 scores available from the data set of [14], written in the standard notation. (ZIP) [file pone.0149688.s001.zip › DATABASE/imgs_SCANED/img023_21.png]

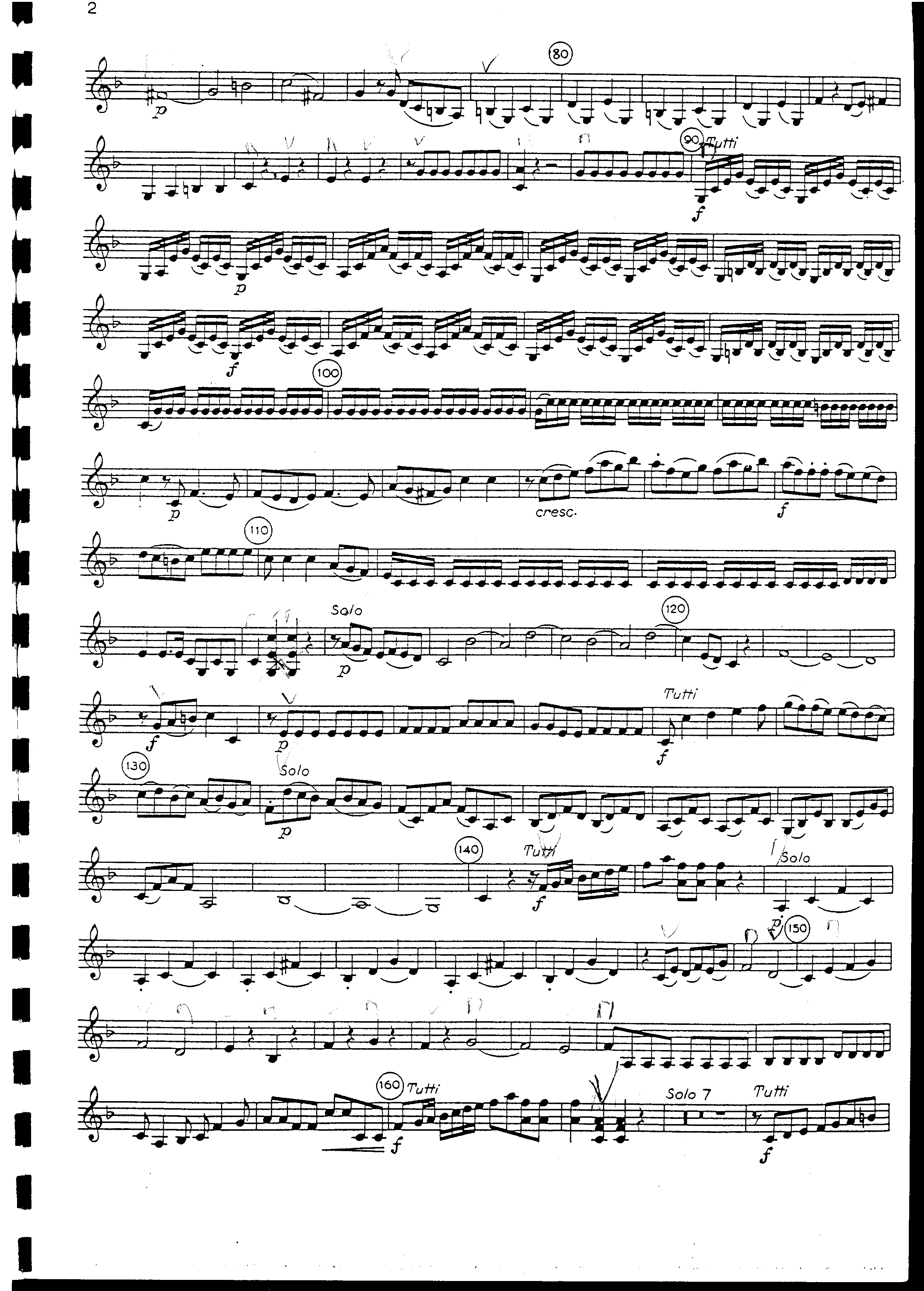

Supplement: S1 File — The real scores consist of 6 handwritten scores from 6 different composers. In the scanned data set, there are 9 scores available from the data set of [14], written in the standard notation. (ZIP) [file pone.0149688.s001.zip › DATABASE/imgs_SCANED/img024_22.png]

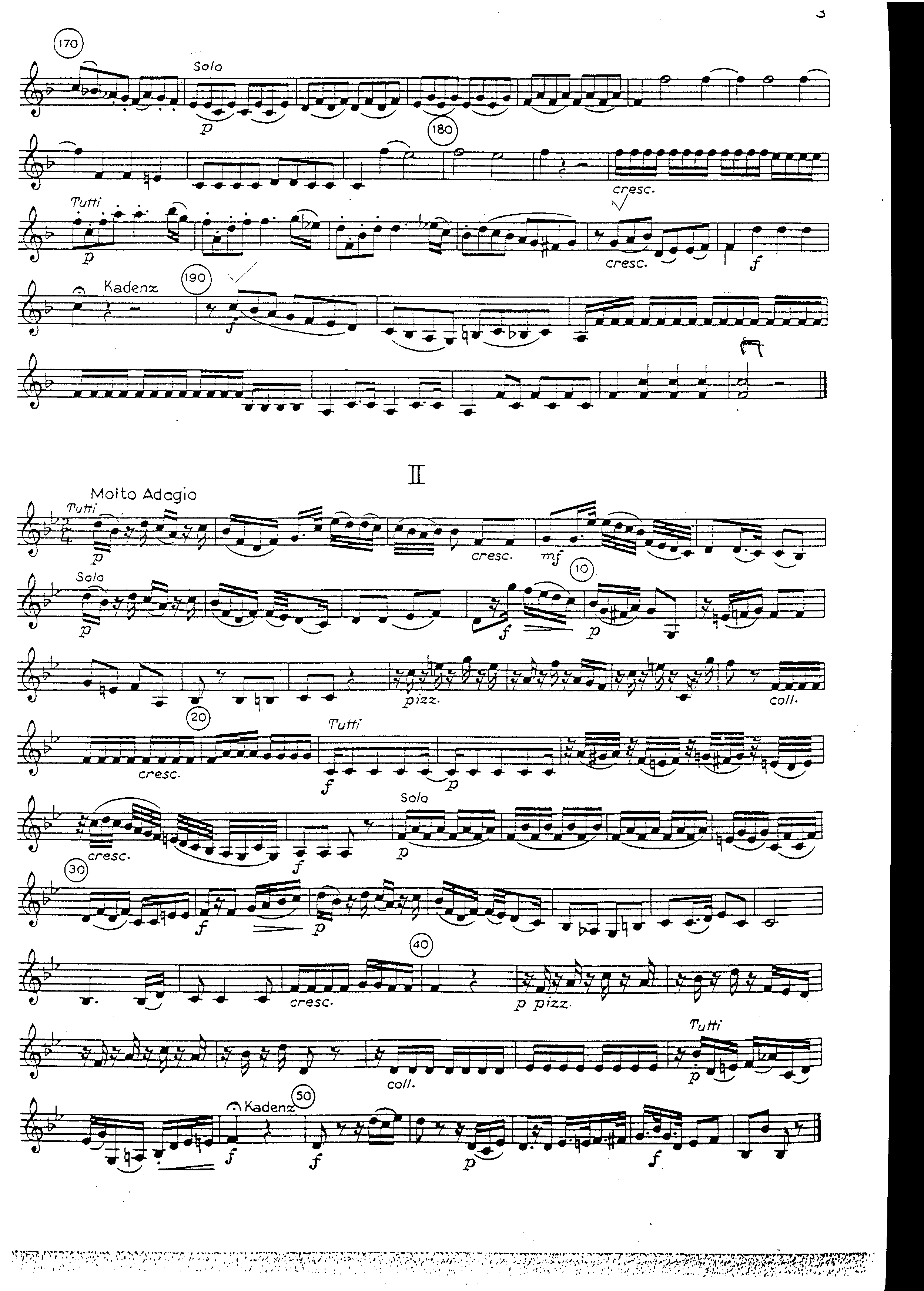

Supplement: S1 File — The real scores consist of 6 handwritten scores from 6 different composers. In the scanned data set, there are 9 scores available from the data set of [14], written in the standard notation. (ZIP) [file pone.0149688.s001.zip › DATABASE/imgs_SCANED/img025_23.png]

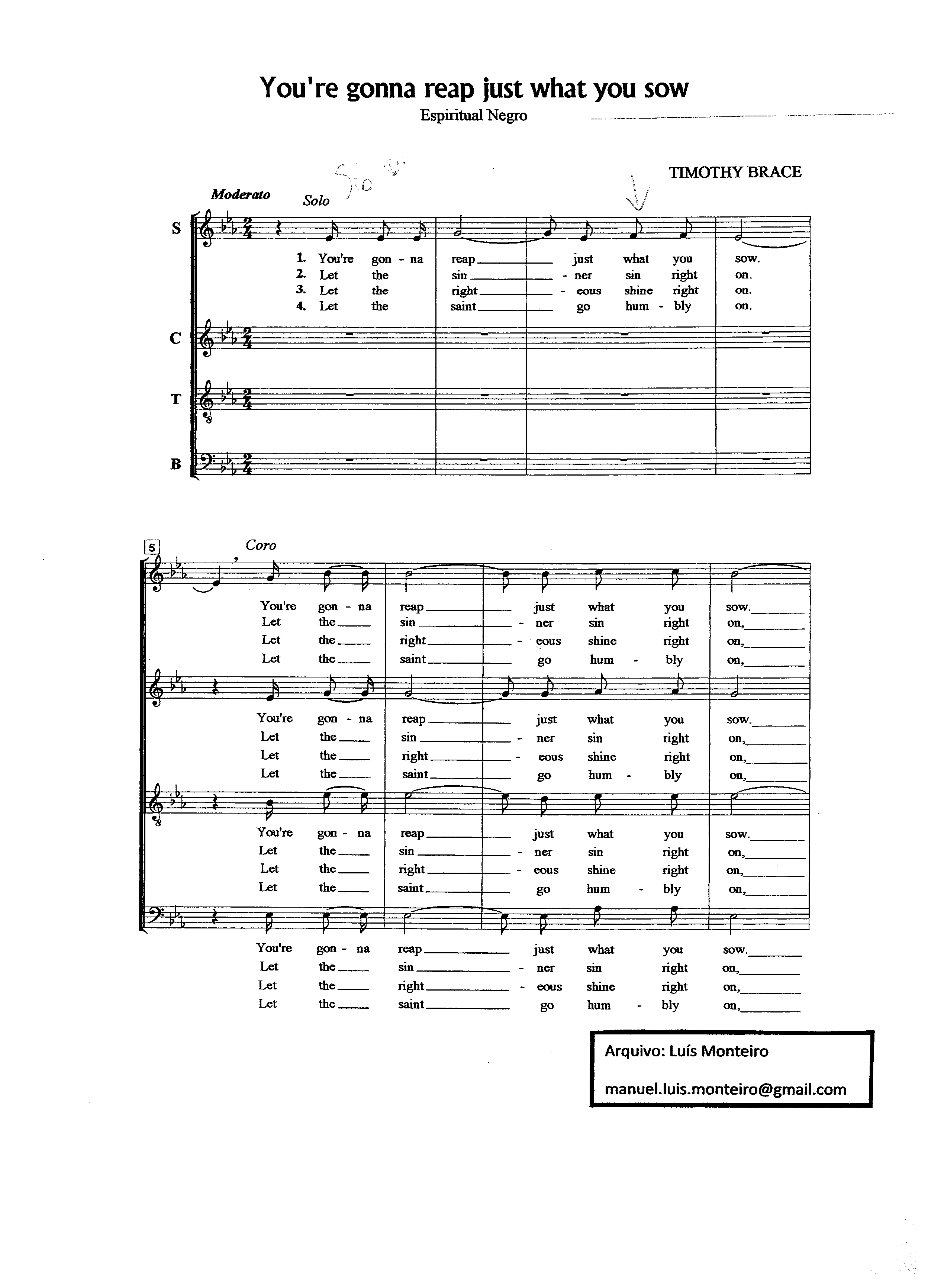

Supplement: S1 File — The real scores consist of 6 handwritten scores from 6 different composers. In the scanned data set, there are 9 scores available from the data set of [14], written in the standard notation. (ZIP) [file pone.0149688.s001.zip › DATABASE/imgs_SCANED/img028.png]

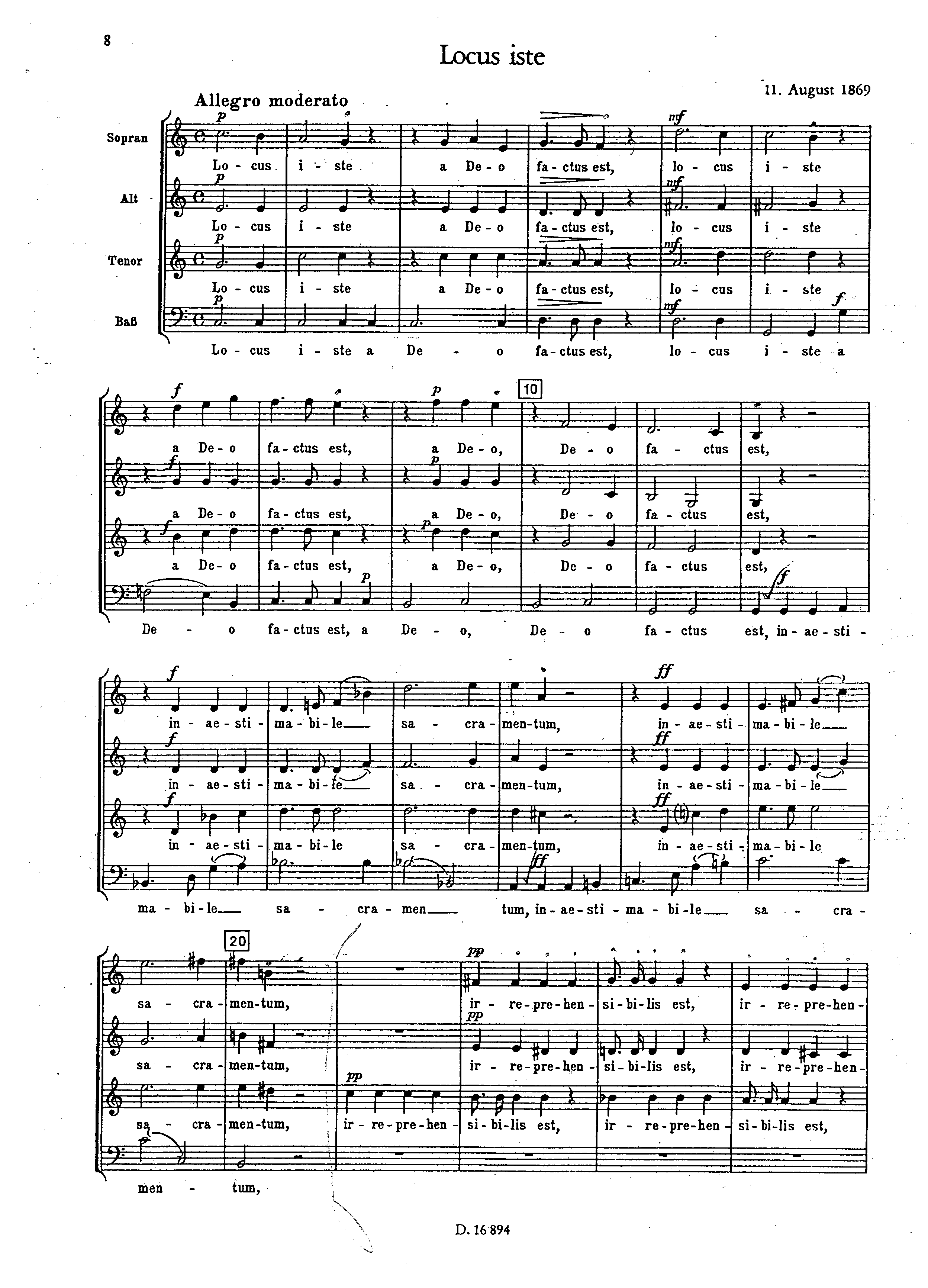

Supplement: S1 File — The real scores consist of 6 handwritten scores from 6 different composers. In the scanned data set, there are 9 scores available from the data set of [14], written in the standard notation. (ZIP) [file pone.0149688.s001.zip › DATABASE/imgs_SCANED/img043.png]

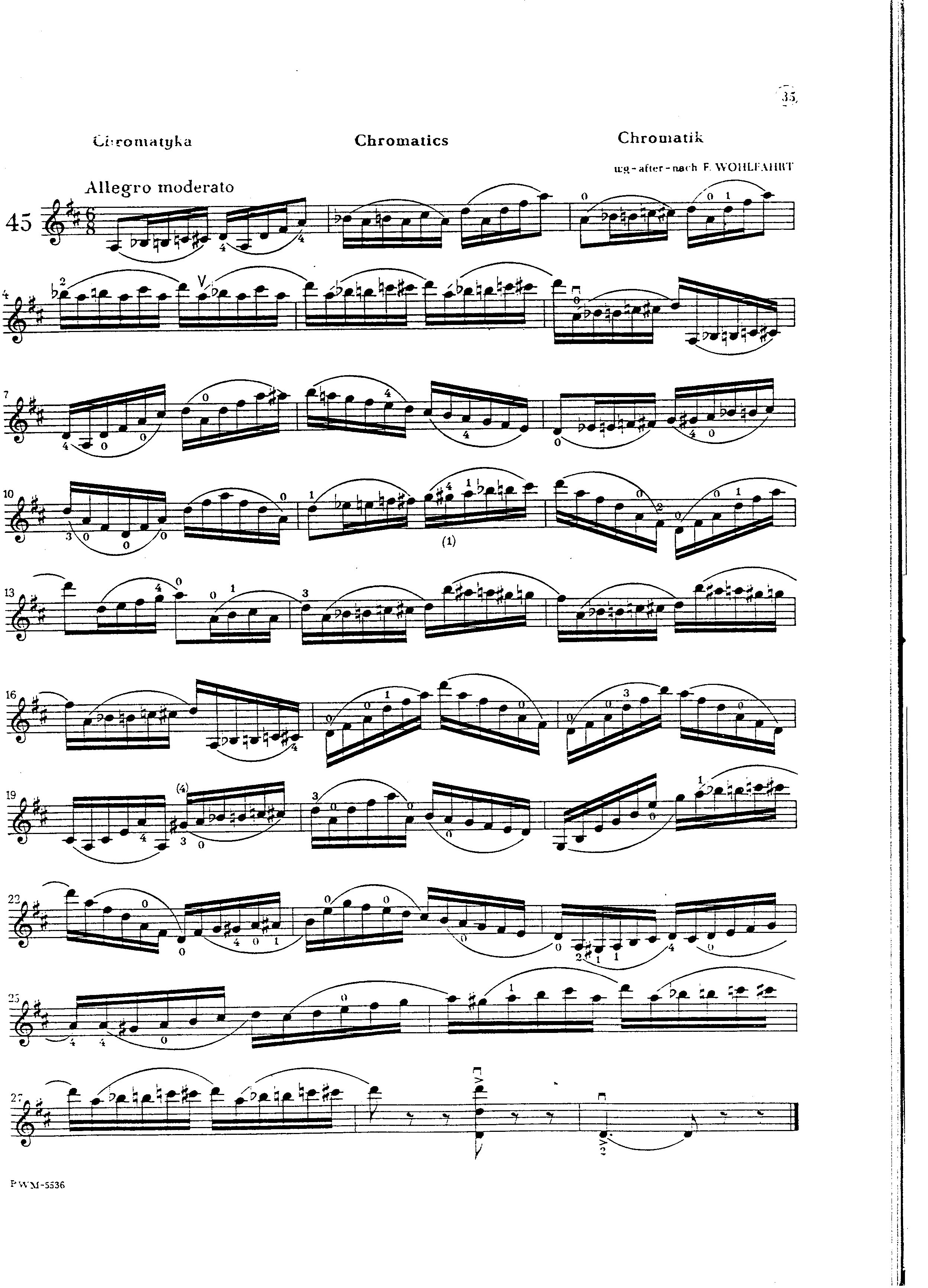

Supplement: S1 File — The real scores consist of 6 handwritten scores from 6 different composers. In the scanned data set, there are 9 scores available from the data set of [14], written in the standard notation. (ZIP) [file pone.0149688.s001.zip › DATABASE/imgs_SCANED/img058.png]

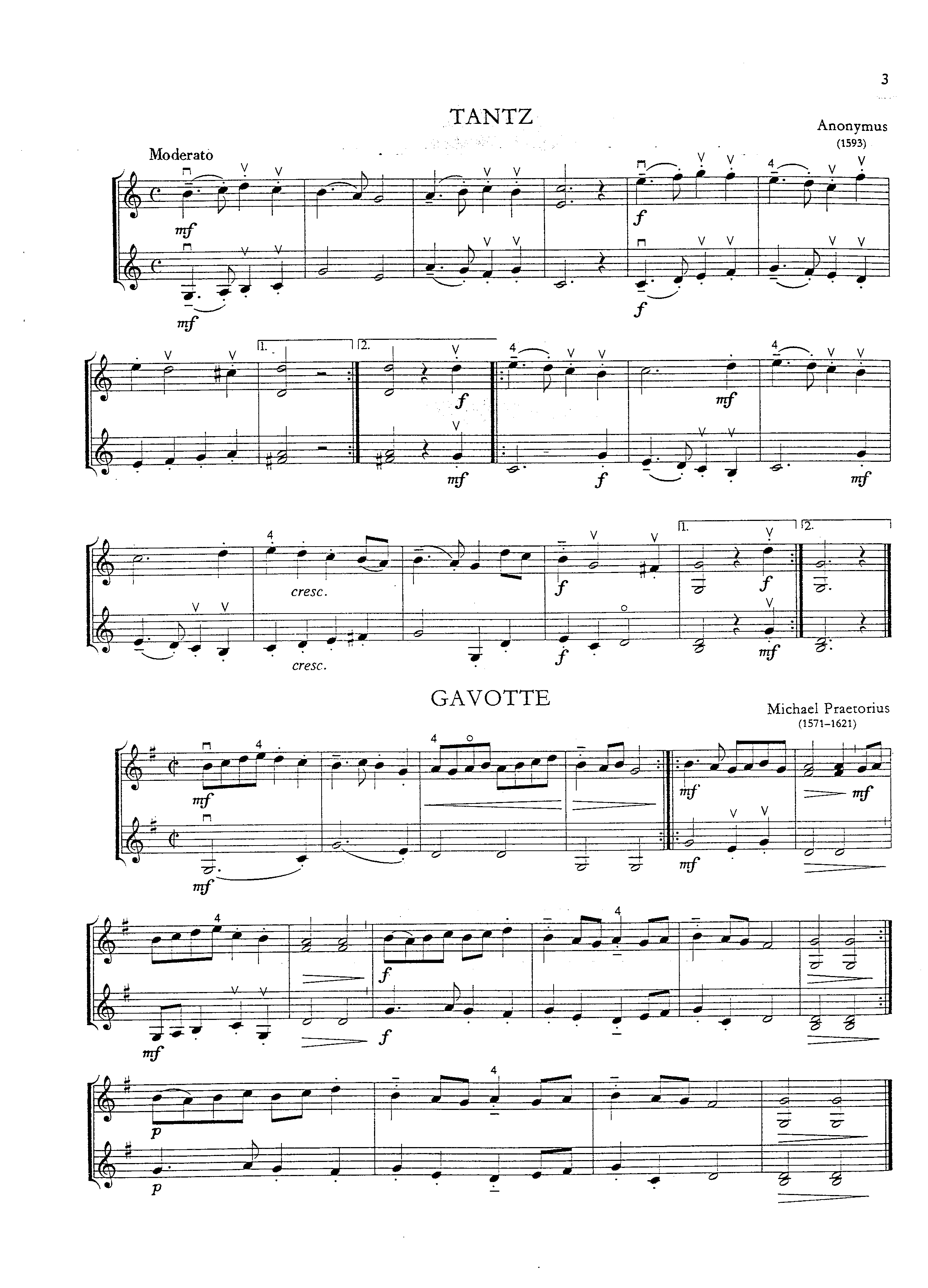

Supplement: S1 File — The real scores consist of 6 handwritten scores from 6 different composers. In the scanned data set, there are 9 scores available from the data set of [14], written in the standard notation. (ZIP) [file pone.0149688.s001.zip › DATABASE/imgs_SCANED/img065.png]
